# Long-term disease control by trabectedin in a patient with dedifferentiated liposarcoma: A case report

**DOI:** 10.1097/MD.0000000000018689

**Published:** 2020-01-10

**Authors:** Shogo Matsuda, Kazuhiro Tanaka, Masanori Kawano, Tatsuya Iwasaki, Ichiro Itonaga, Hiroshi Tsumura

**Affiliations:** Department of Orthopaedic Surgery, Faculty of Medicine, Oita University, Oita, Japan.

**Keywords:** liposarcoma, soft tissue sarcoma, trabectedin

## Abstract

**Rationale:**

Trabectedin is an antitumor drug considered to be effective for liposarcoma, leiomyosarcoma, and translocation-related sarcoma. Concerning liposarcoma subtypes, evidence of the efficacy of trabectedin for dedifferentiated liposarcoma (DDLPS) is poor, whereas it is known to have high efficacy against myxoid liposarcoma. Moreover, there are few reports of long-term trabectedin treatment of cases of DDLPS. Here, we present a case of advanced metastatic DDLPS that achieved long-term disease control by trabectedin treatment.

**Patient concerns:**

A 68-year-old man presented with a mass in his back. Magnetic resonance imaging showed a tumorous mass in his back which exhibited low intensity on T1-weighted and high intensity on T2-weighted images.

**Diagnosis:**

The mass was diagnosed as DDLPS by open biopsy.

**Interventions:**

The patient underwent gemcitabine+docetaxel combination therapy followed by pazopanib and eribulin; all these therapies failed to halt disease progression. Subsequently, we changed the regimen to trabectedin as fourth-line therapy.

**Outcome:**

The patient achieved stable disease for approximately 18 months during 11 cycles of trabectedin therapy.

**Lessons:**

Trabectedin should be considered as a treatment option for DDLPS even in cases of numerous failed prior chemotherapy regimens.

## 1 Introduction

Trabectedin, a tetrahydroisoquinoline alkaloid isolated from the marine ascidian, binds to the minor groove of DNA and affects the DNA repair mechanism resulting in the inhibition of cell proliferation and induction of cell apoptosis.^[1]^ In 2007, the European Medical Agency approved trabectedin for treating advanced soft tissue sarcoma (STS) after previous treatment. Trabectedin was also approved in Japan and the United States in 2015.

Trabectedin is an effective chemotherapeutic drug for liposarcoma, leiomyosarcoma (i.e., L-sarcoma), and translocation-related sarcoma (TRS)^[2–5]^ and is usually used as second-line or later treatment. In terms of the subtypes of liposarcoma, evidence of the efficacy of trabectedin for dedifferentiated liposarcoma (DDLPS) is poor, whereas it is known to have high efficacy against mucinous liposarcoma.^[2]^ Moreover, there are few reports of long-term trabectedin treatment of patients with DDLPS. Here, we present a case of long-term disease control of metastatic DDLPS achieved by trabectedin treatment after a number of failed prior chemotherapies.

## 2 Case report

**Figure 1:**
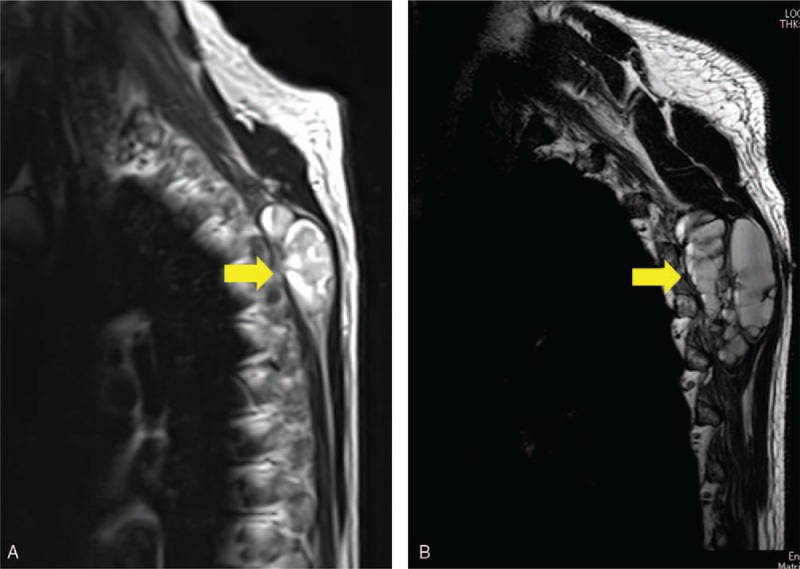
MRI. (A) MRI showing a tumorous mass of 54 × 43 × 30 mm in the latissimus muscle of the back (arrow). (B) After 2 cycles of GD therapy, MRI showed that the size of the back mass was increasing (arrow). GD = gemcitabine+docetaxel, MRI = magnetic resonance imaging.

**Figure 2:**
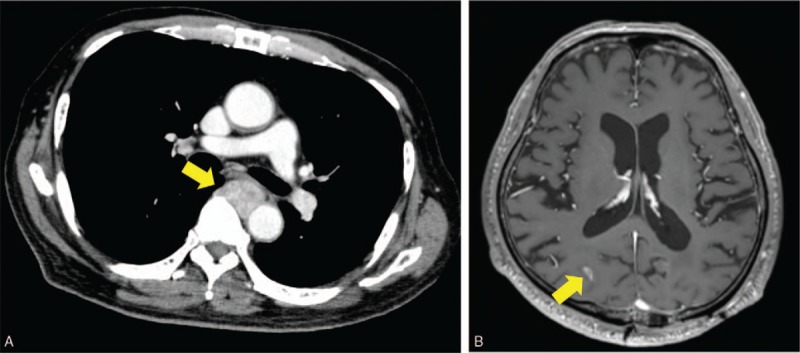
CT of the thorax and MRI of the head after 2 cycles of GD therapy. (A) CT showing lymph node metastasis around the artery (arrow). (B) MRI showing metastasis at the right occipital lobe (arrow). CT = computed tomography, GD = gemcitabine+docetaxel, MRI = magnetic resonance imaging.

**Figure 3:**
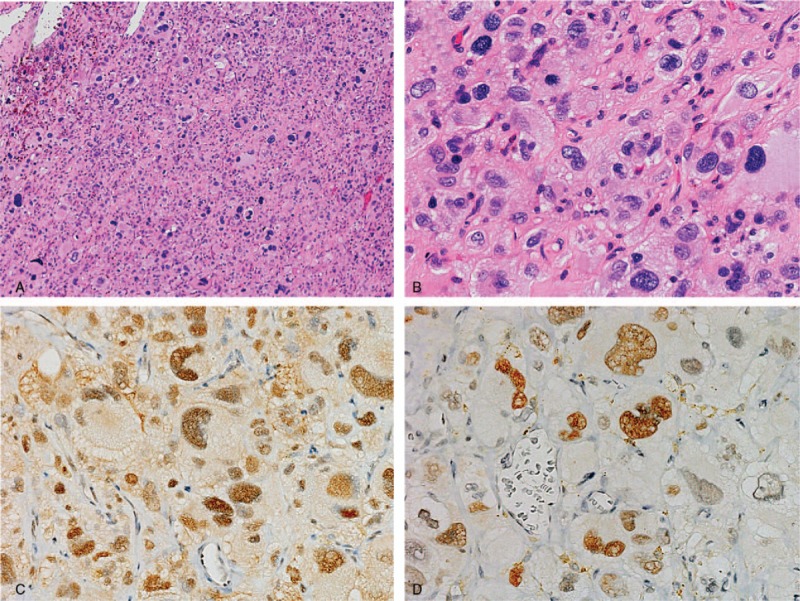
Pathological findings. Hematoxylin and eosin staining at 100× (A) and 200× (B) magnification. Immunohistochemistry of MDM2 (C) and CDK4 (D).

A 68-year-old man presented with a mass in his back. He had a history of atrial fibrillation, diabetes mellitus, and a brain tumor which was completely resected. He was admitted to hospital in May 2015 because of the increasing size of the back mass. On radiograph, the mass was suspected to be STS, and he was referred to our hospital in August 2015. We palpated the elastic soft tumor in his back. X-ray showed no abnormality in the bone. Magnetic resonance imaging (MRI) showed a tumorous mass of 54 × 43 × 30 mm in the latissimus muscle of the back which exhibited low intensity on T1-weighted and high intensity on T2-weighted images (Fig. 1). On computed tomography (CT) of the thorax and abdomen and a head MRI, there was no distant metastasis. After an open biopsy, we diagnosed the mass as DDLPS. Because of the patient's history of heart disease, we avoided the use of doxorubicin, a standard chemotherapeutic agent for DDLPS. Instead, gemcitabine+docetaxel (GD) combination therapy was administered as the first-line treatment. After 2 cycles of GD treatment, however, MRI showed that the mass was increasing in size. Furthermore, abdominal CT and head MRI revealed lymph node and brain metastases, respectively (Fig. 2). To reduce the symptoms caused by the local disease, we performed a wide resection of the back mass. Pathologic diagnosis of the resected specimen was also DDLPS with positive immunohistochemical staining for murine double minute 2 (MDM2) and cyclin-dependent kinase 4 (CDK4) (Fig. 3).

**Figure 4:**
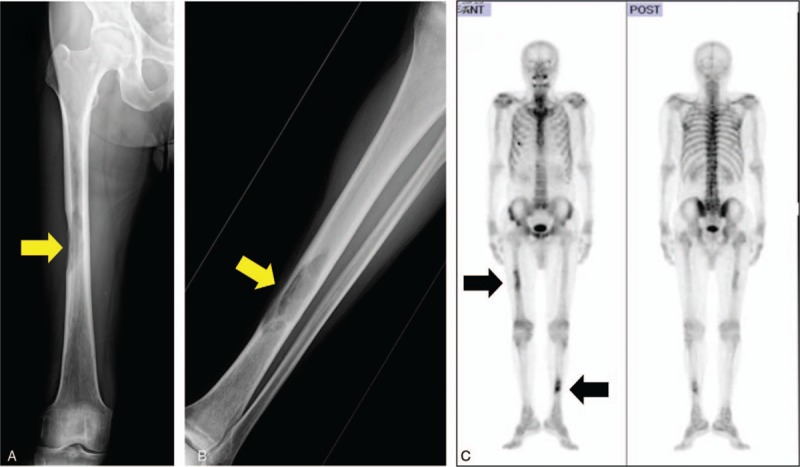
X-ray and bone scintigraphy after dosing with pazopanib. X-ray showing a radiolucent shadow at the right femur diaphysis (A, arrow) and left tibia diaphysis (B, arrow). (C) Bone scintigraphy showing metastases at the right femur diaphysis and left tibia diaphysis (arrows).

**Figure 5:**
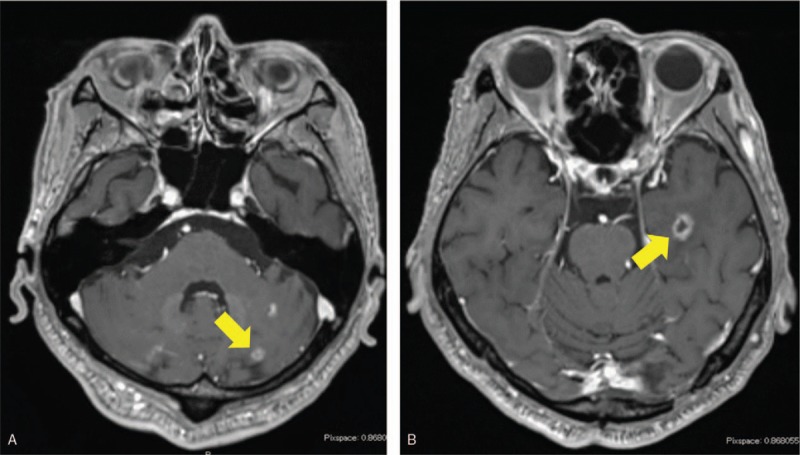
Head MRI after dosing with eribulin. MRI showing new metastases in the left cerebellar hemisphere (A, arrow) and left temporal lobe (B, arrow) after eribulin treatment. MRI = magnetic resonance imaging.

**Figure 6:**
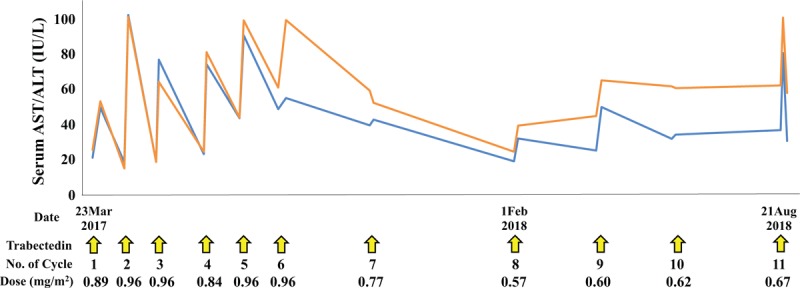
Trabectedin administration period and dose modification, and changes in serum transaminases (blue line, AST; orange line, ALT). ALT = alanine aminotransferase, AST = aspartate aminotransferase.

**Figure 7:**
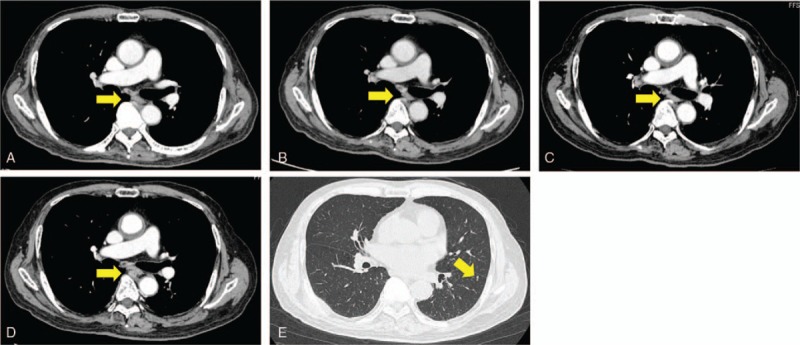
CT of the thorax after dosing with trabectedin. (A) April 2017. (B) November 2017. (C) May 2018. (D) September 2018 after 11 cycles of trabectedin. No significant change was observed in the lymph node metastasis around the artery (arrow). (E) CT showing new lung metastasis at the left inferior lobe (arrow). CT = computed tomography.

After the surgery, the chemotherapy regimen was changed to pazopanib at a dose of 600 mg/d, and Gamma Knife treatment was performed for the brain metastasis. Pazopanib induced an elevation in serum transaminases (Grade 2); thus, we reduced the dose of pazopanib to 200 mg/d. However, bone metastases were found in the left tibia and right femur (Fig. 4). Subsequently, we performed wide resection of the metastatic tumors in the left tibia and right femur and reconstruction with liquid nitrogen-treated bone. We switched the chemotherapy regimen to eribulin; however, we found a further recurrence of brain metastasis and disease progression after 6 cycles of eribulin treatment (Fig. 5). We performed CyberKnife treatment for the brain metastasis. Finally, we used trabectedin (1.2 mg/m^2^) as the fourth-line of chemotherapy with central venous catheter. Firstly, we administered trabectedin (0.89 mg/m^2^) because we concerned about adverse events. Overall, 11 cycles of trabectedin were performed with decreasing dosages due to adverse events, including hypophagia and elevation of transaminases. The frequency of trabectedin, dosage alteration, and changes in serum transaminases were shown in Fig. 6. Stable disease (SD) was achieved for 18 months during trabectedin treatment until termination of the treatment as a result of a new metastasis in the lung (Fig. 7). The patient was then provided with best supportive care. The patient has provided informed consent for publication of the case.

## 3 Discussion

The standard first-line chemotherapy for advanced STS is a doxorubicin-based regimen.^[6]^ In contrast, there are many options of chemotherapy for the second-line, including GD, pazopanib, eribulin, and trabectedin. However, there is no evidence to indicate which regimen is optimal for second-line or later treatment.^[6]^ Therefore, drug selection is done by considering the histological type, organ function, performance status, and comorbidity for each case.

Trabectedin is a tetrahydroisoquinoline alkaloid derived from the Caribbean tunicate, *Ecteinascidia turbinate*. Trabectedin binds to miner groove of DNA and affects DNA repairing mechanism, subsequently inhibits cell proliferation and induces cell apoptosis.^[1]^ Trabectedin also influences anti-inflammatory and immunomodulatory properties. The efficacy of trabectedin on STS might be related to a molecular targeting mechanism to the fusion gene products in translocation-positive STS.^[5]^ The standard dose of trabectedin is 1.5 mg/m^2^ in the United States and Europe, however, the approved dose of trabectedin is 1.2 mg/m^2^ in Japan because of toxicities in Japanese patients.^[3]^ Samuels et al^[7]^ reported that overall survival (OS) of patients with metastatic or unresectable STS treated with trabectedin as the second-line therapy was better in patients with L-sarcoma than in those with other types of STS. In a phase III trial of trabectedin versus doxorubicin-based chemotherapy as first-line treatment for TRS, trabectedin improved progression-free survival (PFS) and OS, although there was no statistical difference between the 2 treatment groups.^[4]^ In another phase III trial of trabectedin versus dacarbazine chemotherapy for L-sarcoma, the trabectedin arm achieved significantly greater rates of PFS. Moreover, in a subgroup analysis of liposarcoma, there was a statistical improvement in PFS of patients with myxoid liposarcoma, but no significant difference in survival between the 2 treatment groups of DDLPS and pleomorphic liposarcoma.^[2]^ Considering the above results, the efficacy of trabectedin for DDLPS is of great concern. Therefore, we first administrated GD followed by pazopanib and eribulin in the present case. After disease progression despite the intensive prior treatments, trabectedin was administrated, unexpectedly resulting in long-term disease control for about 18 months. Since trabectedin showed the tendency of better OS and PFS in the phase III trial of the first-line treatment for STS,^[4]^ we consider that the efficacy might have been greater if we had administrated trabectedin as first-line, and not as fourth-line, treatment for chemotherapy-refractory DDLPS.

Only 2 case reports of long-term administration of trabectedin have been published. Kus et al^[8]^ reported a case that underwent 6 cycles of trabectedin as second-line therapy for metastatic DDLPS that achieved complete remission. Haslbauer^[9]^ reported a long-term response in a 71-year-old patient with metastatic leiomyosarcoma who received 22 cycles of trabectedin as second-line treatment and successfully maintained SD for 2 years.^[8]^ However, there is no previous report of a case of DDLPS that achieved SD for as long as 18 months with 11 cycles of trabectedin as fourth-line chemotherapy.

In conclusion, we report, for the first time, a rare case of metastatic DDLPS that achieved long-term SD for a period of 18 months through 11 cycles of trabectedin after 3 prior chemotherapy regimens. Trabectedin might be an important treatment option for DDPLS because it has the potential to achieve long-term disease control of DDLPS after several failed prior regimens.

## Author contributions

**Conceptualization:** Shogo Matsuda, Kazuhiro Tanaka, Masanori Kawano, Tatsuya Iwasaki, Ichiro Itonaga, Hiroshi Tsumura.

**Data curation:** Shogo Matsuda, Kazuhiro Tanaka, Masanori Kawano, Tatsuya Iwasaki, Ichiro Itonaga, Hiroshi Tsumura.

**Funding acquisition:** Kazuhiro Tanaka.

**Investigation:** Shogo Matsuda, Masanori Kawano, Tatsuya Iwasaki, Ichiro Itonaga, Hiroshi Tsumura.

**Supervision:** Kazuhiro Tanaka.

**Writing – original draft:** Shogo Matsuda, Kazuhiro Tanaka, Masanori Kawano, Tatsuya Iwasaki, Ichiro Itonaga, Hiroshi Tsumura.

**Writing – review & editing:** Shogo Matsuda, Kazuhiro Tanaka, Masanori Kawano, Tatsuya Iwasaki, Ichiro Itonaga, Hiroshi Tsumura.
